# Long non-coding RNA-encoded micropeptides: functions, mechanisms and implications

**DOI:** 10.1038/s41420-024-02175-0

**Published:** 2024-10-23

**Authors:** Yinan Xiao, Yaru Ren, Wenteng Hu, Athanasios R. Paliouras, Wenyang Zhang, Linghui Zhong, Kaixin Yang, Li Su, Peng Wang, Yonghong Li, Minjie Ma, Lei Shi

**Affiliations:** 1 RNA Oncology Group, School of Public Health, Lanzhou University, Lanzhou, 730000 PR China; 2 Thoracic surgery department, The First Hospital, Lanzhou University, Lanzhou, 730000 PR China; 3 Celeritas Insights 13, Freeland Park, Poole, BH16 6FA UK; 4 College of Animal Science and Technology, Hebei North University, Zhangjiakou, 075131 PR China; 5 NHC Key Laboratory of Diagnosis and Therapy of Gastrointestinal Tumor, Gansu Provincial Hospital, Lanzhou, 730000 PR China

**Keywords:** Cancer genomics, Cancer epigenetics

## Abstract

Long non-coding RNAs (lncRNAs) are typically described as RNA transcripts exceeding 200 nucleotides in length, which do not code for proteins. Recent advancements in technology, including ribosome RNA sequencing and ribosome nascent-chain complex sequencing, have demonstrated that many lncRNAs retain small open reading frames and can potentially encode micropeptides. Emerging studies have revealed that these micropeptides, rather than lncRNAs themselves, are responsible for vital functions, including but not limited to regulating homeostasis, managing inflammation and the immune system, moderating metabolism, and influencing tumor progression. In this review, we initially outline the rapidly advancing computational analytical methods and public tools to predict and validate the potential encoding of lncRNAs. We then focus on the diverse functions of micropeptides and their underlying mechanisms in the pathogenesis of disease. This review aims to elucidate the functions of lncRNA-encoded micropeptides and explore their potential applications as therapeutic targets in cancer.

## Facts

- LncRNAs play an essential role in diverse biological manners.
- LncRNAs can encode micropeptides.
- LncRNA-encoded micropeptides affect human innate immunity, metabolism, tumorigenesis.

## Questions

- Which is the best method to identify the lncRNA-encoded micropeptides?
- What is the physiological function of lncRNA-encoded micropeptides?
- What is the underlying mechanism of bi-functional lncRNAs, either as coding peptides or ncRNA molecules, in human diseases?

## Introduction

The Central Dogma of molecular biology posits that genetic information, encapsulated within genes as either DNA or RNA sequences, is translated into functional products, predominantly proteins [1]. Emerging advancements in next-generation sequencing technologies over the past two decades have significantly deepened our understanding of the transcriptome providing novel insights into the genetic orchestra. Astonishingly, it appears that up to 98% of RNA transcripts within the human genome are non-coding RNAs (ncRNAs), which do not code for proteins [2]. These non-coding RNAs have been referred to as “noise DNA” or “dark matter” since they were once believed to be worthless parts of the genome. However, recent research has brought attention to these hitherto overlooked molecular actors, illuminating the crucial regulatory roles of ncRNAs in a spectrum of fundamental biological processes—from metabolism to development and differentiation [3]. According to their size, ncRNAs can be broadly divided into different clusters, such as microRNAs, circular RNAs (circRNAs), long non-coding RNAs (lncRNAs), PIWI-interacting RNAs (piRNAs) and snoRNAs [4]. ncRNAs are involved in most human physiological diseases [5]. Such revelations underscore the potential of ncRNAs not only as diagnostic markers but also as targets for therapeutic intervention.

LncRNAs are a class of ncRNAs longer than 200 nucleotides. In general, lncRNAs are transcribed like messenger RNAs by the RNA polymerase II, capped at the 5’end, polyadenylated at the 3’end, and spliced [6]. Distinct from mRNAs, lncRNAs exhibit tissue-specific expression and directly modulate a plethora of biological processes. They exert diverse functions, including microRNA sponge, RNA stabilization, transcription regulation, and remodeling chromatin and genome architecture [7]. Numerous studies have explored the diverse and significant roles of lncRNAs in cancer development, where they can act as oncogenes, tumor suppressors, and chromatin scaffolds [8]. Recently, scientists have become aware that lncRNAs carry small open reading frames (sORFs) and encode micropeptides [9]. An instance of this includes the work by Huang and colleagues, who discovered that the lncRNA HOXB-AS3 produces a 53-amino acid peptide named HOXB-AS3. This peptide inhibits colon cancer (CRC) growth by binding with high affinity to the arginine residue motif of hnRNP A1, which impedes the splicing of pyruvate kinase M (PKM) by hnRNP A1 [10]. In another intriguing example, Ge and team identified a 94 amino acid-length micropeptide called the ATP synthase–associated peptide (ASAP), which is encoded by the lncRNA LINC00467. They showed that ASAP interacts with ATP synthase subunits α and γ (ATP5A and ATP5C), facilitating ATP synthase assembly, which boosts its activity and mitochondrial oxygen consumption. This results in augmented colorectal cancer cell proliferation [11].

In this review, we outline the rapidly advancing field of lncRNA-encoded proteins, encompassing both computational methodologies and their biological significance. We draw attention to the fact that certain lncRNA-encoded functional peptides with relevance to cancer play a central role in regulating various biological processes, and influence tumor initiation, progression, invasion, and metastasis. We outline the future outlook on the current research landscape of lncRNA-encoded micropeptides in therapy, aiming to provide novel implications and strategies in cancer.

## Molecular functions of lncRNAs

**Fig. 1:**
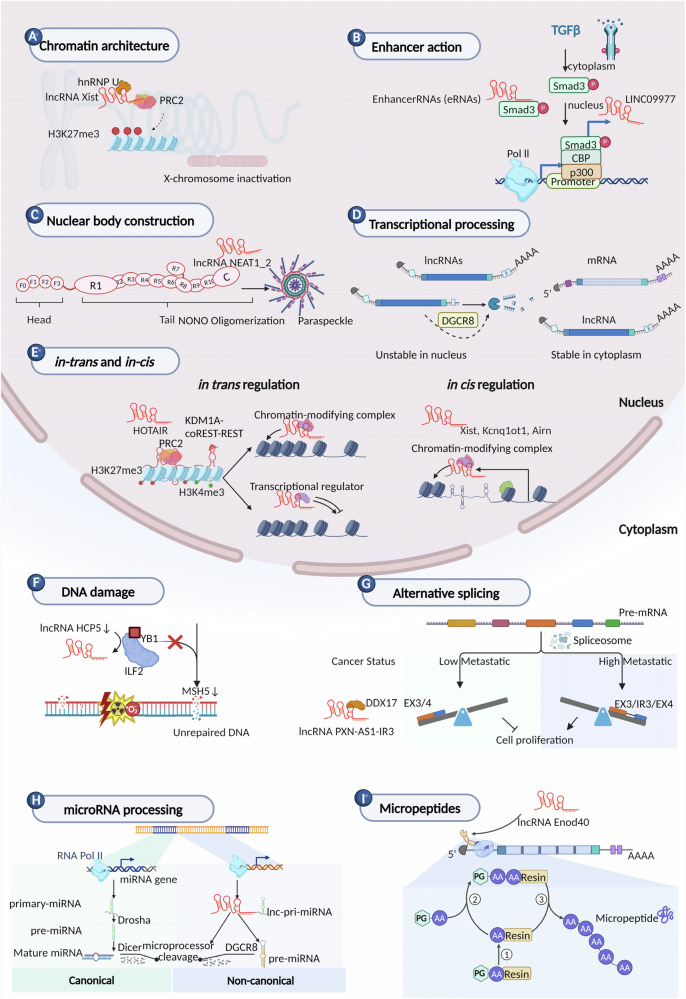
Diverse functions of lncRNAs. **A** LncRNA XIST alters three-dimensional genome architecture during X-chromosome inactivation. **B** LncRNA LINC09977 functions as an enhancer to stimulate the TGFβ/SMAD3 pathway. **C** NEAT1_2 recruits NONO dimers that initiate paraspeckle assembly. **D** The involvement of lncRNA in transcriptional processing via the microprocessor complex subunit DGCR8. **E** LncRNA can modulate gene expression in regions distant (in trans) or nearby (*in cis*) from their transcription sites. **F** LncRNA HCP5 affects DNA damage via YB1-mediated MSH5 in the nucleus. **G** Enforced DDX17 activates tumorigenesis by producing a long-spliced transcript of lncRNA PXN-AS1-IR3. **H** Microprocessor genes cleave the nascent transcript lnc-pri-miRNAs and result in microRNA processing in a noncanonical pathway. **I** LncRNA encoded micropeptide ENDO40 is involved in root organogenesis.

LncRNAs are a class of RNAs that are affecting a large number of biological processes. These include but are not limited to influencing chromatin architecture, enhancing action, contributing to the phase separation, engaging in transcription processing, and exerting both in-trans and *in-cis* regulatory functions. Additionally, lncRNAs are involved in alternative splicing, DNA damage repair, microRNA processing, and even encoding micropeptides. Each of these aspects underscores the versatile and pivotal nature of lncRNAs within cellular biology (Fig. 1) [12]. (A) Chromatin architecture: Engreitz and colleagues marked a significant observation with lncRNA XIST, showcasing its ability to cover the entire X chromosome by leveraging its spatial proximity to 3D conformation during X chromosome inactivation (XCI), exemplifying the critical role in chromatin reorganization (Fig. 1A) [13]. (B) Enhancer action: Zhang and colleagues showed that M2-like tumor-associated macrophages (TAM2) infiltration facilitates a rich TGFβ microenvironment and promotes SMAD3 binding to the enhancer of Linc01977, therefore initiating malignancy through the TGFβ/SMAD3 pathway in lung cancer (Fig. 1B) [14]. (C) Nuclear body construction: Yamazaki reported that NEAT1_2 middle subdomains recruit NONO dimers that initiate paraspeckle assembly with phase-separated features (Fig. 1C) [15]. Xing identified that a snoRNA-end lncRNA SLERT binds to RNA helicase DDX21 RecA domain, in order to control fibrillar center and the dense fibrillar component (DFC) phase separation and reshapes the donut-like ring structures, therefore preventing the repression of PoI I transcription [16, 17]. (D) Transcriptional processing: Schlackow used mNET-seq to survey genome-wide PoI II density and found a different phosphorylation status of the Pol II C-terminal domain (CTD) between mRNAs and lncRNAs. LncRNAs are inefficiently polyadenylated and spliced and more degraded post-transcriptionally by the nuclear exosome (Fig. 1D) [18]. (E) in-trans and in-cis regulation: LncRNAs’ regulatory roles can be generally categorized into in-trans and *in-cis* regulation. In-trans regulatory lncRNAs modulate gene expression in regions distant from their transcription sites via influencing chromosome structure and the interacting proteins or RNA molecules [19]. In addition to *in* *trans-acting*, *in cis-acting* lncRNA molecules can recruit other proteins or complexes to nearby loci in order to modulate gene activity (Fig. 1E) [19]. (F) DNA damage: Wang recently reported that the lncRNA HCP5 could interact with YB1 and ILF2, therefore resulting in the shuttling of YB1 to the nucleus to stimulate MSH5 and affect DNA damage repair (Fig. 1F) [20]. (G) Alternative splicing: Zhou demonstrated that an intron 3 retention transcript of lncRNA PXN-AS1 (PXN-AS1-IR3) recruits p300 to the MYC promoter, activating MYC downstream genes and facilitating hepatocellular carcinoma (HCC) metastasis (Fig. 1G) [21]. (H) microRNA processing: Some lncRNAs are the host genes of microRNAs and do not apply to the canonical cleavage-and-polyadenylation pathway. Microprocessors (Dicer, DGCR8 and others) cleave the nascent transcript lnc-pri-miRNAs rather than in the typical polyadenylation-dependent manner (Fig. 1H) [22, 23]. (I) Micropeptides: Rohrig reported that ENOD40, a plant long noncoding RNA enod40, can encode functional peptides, in the case of a sucrose-synthesizing enzyme during root organogenesis, vividly illustrates the coding potential latent within ncRNAs (Fig. 1I) [24, 25]. In summary, lncRNAs are not mere passengers but active and versatile conductors of a multitude of cellular and biological processes, heralding a new era of understanding the complexity and elegance of RNA-mediated regulation.

## Prediction and identification of lncRNA-encoded micropeptides

In recent years, significant advancements have been made in methods to explore the coding capacity and potential functions of micropeptides. These approaches encompass a range of techniques, including predicting ORFs, analyzing translation start elements such as internal ribosome entry sites (IRES), investigating histone modifications, conducting translation omics and proteomics profiling, and utilizing Flag-labeled expression combined with mass spectrometry (Table 1) [9].

**Table 1:** Prediction tools, identification tools, and databases for lncRNA-encoded micropeptides.

Method	Description	Website	Ref.
**Open reading frame Analysis**			
ORF finder	Web server for identifying ORFs along with the protein translation using newly developed SMART BLAST or regular BLASTP.	https://www.ncbi.nlm.nih.gov/orffinder/	[90]
sORF finder	Identification of the coding potential according to the nucleotide composition bias among coding sequences and the potential functional constraint at the amino acid level.	https://dl.acm.org/doi/10.1093/bioinformatics/btp688	[91]
ORFanage	Use reference annotation to maximize the similarities between identified ORFs and the protein sequence.	https://github.com/alevar/ORFanage	[92]
PhyloCSF	A novel track generated from Broad Institute that aids in identification of functional conserved, protein-coding regions of genomes.	http://compbio.mit.edu/PhyloCSF/	[30, 93]
uPEPperoni	Detect conserved ORFs in eukaryotic transcripts by comparing query nucleotide sequences against mRNA sequences within the NCBI RefSeq database.	http://upep-scmb.biosci.uq.edu.au/	[94, 95]
COME	A computational tool that predicts the coding potential for a given transcript by integrating multiple sequence-derived and experiment-based features.	https://github.com/lulab/COME	[31]
CRITICA	Determination the likely protein-coding sequences in DNA by combining comparative analysis of DNA sequences with more common noncomparative methods.	https://ngdc.cncb.ac.cn/biocode/tools/BT004311	[33]
CPC	Assess the protein-coding potential of a transcript utilizing sequence features and support vector machine (SVM).	http://cpc.gao-lab.org/	[27]
CPC2	A fast and accurate coding potential calculator based on sequence intrinsic features.	http://cpc2.gao-lab.org/	[28]
PORTRAIT	Prediction of transcriptomic ncRNA by ab initio methods and provides a low computational cost solution for ncRNA detection in transcriptome sequencing projects.	https://www.bioinformatics.org/portrait/	[96]
CPAT	CPAT uses a logistic regression model built with open reading frame size, open reading frame coverage, Fickett TESTCODE statistic and hexamer usage bias to predict the coding potential of ncRNAs	http://code.google.com/p/cpat/	[26]
CNIT	CNIT provides faster and more accurate evaluation of the coding ability of RNA transcripts.	http://cnit.noncode.org/CNIT	[29]
**IRES prediction**			
IRESite	Curate experimental evidence of many eukaryotic viral and cellular IRES regions.	http://www.iresite.org	[97]
IRESpy	A publicly available tool for all IRES site prediction.	https://irespy.shinyapps.io/IRESpy/	[98]
IRESbase	Curate the experimentally validated IRES elements from literature and annotating their host linear and circular RNAs.	http://reprod.njmu.edu.cn/cgi-bin/iresbase/index.php	[99]
IRESfinder	A python package to identify the RNA internal ribosome entry site in eukaryotic cell.	https://github.com/xiaofengsong/IRESfinder	[100]
IRESPred	Predict both viral and cellular IRES using SVM.	http://bioinfo.net.in/IRESPred/	[101]
**m**^**6**^**A medication prediction**			
DeepM6ASeq	A deep-learning-based framework to predict m^6^A-containing sequences and visualize saliency map for sequences.	https://github.com/rreybeyb/DeepM6ASeq	[40]
SRAMP	It identifies mammalian m^6^A sites at single-nucleotide resolution and predict structural features around m^6^A site.	http://www.cuilab.cn/sramp	[41]
WHISTLE	Provide a high-accuracy map of the human m^6^A epitranscriptome.	http://whistle-epitranscriptome.com	[102]
TargetM6A	Recognition of N^6^-methyladenosine sites from RNA sequences by position-specific nucleotide predisposition and SVM.	http://csbio.njust.edu.cn/bioinf/	[103]
**Transcriptomic-based method**			
Ribosome profiling	A deep-sequencing-based tool that for the experimental annotation of translated ORFs and discovery of a wide range of new translation products.	NA	[104]
Poly-Ribo seq	A combination of ribosome profiling and polysome to enrich more potent peptides and coding ORFs.	NA	[44]
RiboCode	A very simple but high-quality computational algorithm to identify genome-wide translated ORFs using ribosome-profiling data.	NA	[105]
RNC-sequencing	A method for profiling the RNC Complex using next-generation sequencing to evaluate the translational peptides.	NA	[106]
ORF-RATER	The ORF-RATER pipeline globally evaluates translation of RNA transcripts.	https://github.com/alexfields/ORF-RATER/	[107, 108]
RiboTaper	A new analysis pipeline for Ribosome Profiling experiments, which exploits the triplet periodicity of ribosomal footprints to call translated regions.	https://ohlerlab.mdc-berlin.de/software/RiboTaper_126/	[42]
FLOSS	FLOSS classifies the translation status of individual transcripts and sub-regions.	https://rdrr.io/bioc/ribosomeProfilingQC/man/FLOSS.html	[109]
Trap^Seq^	A novel RNA sequencing-based method (Trap^Seq^) to map gene-trap insertions.	https://www.illumina.com.cn/science/sequencing-method-explorer/kits-and-arrays/trap-seq.html	[110]
**Public databases**			
**Database**	**Description**	**Website**	**Ref**.
sORFs.org	A novel database for sORFs identified using ribosome profiling.	http://www.sorfs.org	[111]
SmProt	SmProt contains features for the collected small proteins on their sequences, genomic locations, tissues/cell lines, assessment reflecting coding potential, function, variants, and related diseases via prediction and verification.	http://bigdata.ibp.ac.cn/SmProt	[112]
OpenProt	A complete and freely accessible set of non-canonical or alternative open reading frames (AltORFs) within the transcriptome of various species, as well as functional annotations of the corresponding protein sequences not found in standard databases.	https://www.openprot.org/	[113]
MetamORF	Provide a repository of unique sORFs in human and mouse genomes with experimental and computational approaches.	https://metamorf.hb.univ-amu.fr/	[114]
SPENCER	A comprehensive database for small peptides encoded by noncoding RNAs derived from 2806 mass spectrometry (MS) datasets from 1007 tumor samples and 719 normal samples across 15 different cancer types.	http://spencer.renlab.org/#/home	[115]
ncEP	A friendly tool includes 74 proteins or peptides, 22 lncRNAs, 11 circRNAs, 9 pri-miRNAs and 37 other ncRNAs across 18 species from more than 50 research articles.	http://www.jianglab.cn/ncEP/	[116]
LncPep	Integrating multiple databases including CPAT, CPC2, m^6^A, Ribo-seq, Pfam and TISs to analyze the coding capacity of 883,804 lncRNAs across 39 species.	http://www.shenglilabs.com/LncPep/	[117]
FuncPEP	FuncPEP contains a fundamental annotation of 112 functional ncRNA-encoded peptides from experimentally validated and functionally characterized datasets.	https://bioinformatics.mdanderson.org/Supplements/FuncPEP/	[118]
cncRNADB	A web-based tool for the analysis of 2600 manually curated entries of cncRNA functions with experimental evidence, involving more than 2,000 RNAs (including over 1300 translated ncRNAs and over 600 untranslated mRNAs) across over 20 species.	http://www.rna-society.org/cncrnadb/	[119]

*ORFanage* Open Reading Frames annotation in spliced genomes, *PhyloCSF* Phylogenetic codon models estimated from genome-wide training data, *uPEPperoni* An online tool for upstream open reading frame location and analysis of transcript conservation, *COME* Coding potential calculator based on multiple evidences, *CRITICA* Coding Region Identification Tool Invoking Comparative Analysis, *CPC* Coding Potential Calculator, *PORTRAIT* Prediction of transcriptomic ncRNA by ab initio methods, *CPAT* Coding Potential Assessing Tool, *CNIT* Coding-Non-Coding Identifying Tool, *IRES* Internal Ribosome Entry Site, *DeepM6ASeq* A deep-learning-based framework for studying m6A, *SRAMP* Sequence-based RNA adenosine methylation site predictor, *RNC* Ribosome Nascent-chain Complex, *ORF-RATER* Open Reading Frame - Regression Algorithm for Translational Evaluation of Ribosome-protected footprints, *FLOSS* Fragment length organization similarity score, *Trap*^*Seq*^ Targeted purification of polysomal mRNA, *sORFs* short open reading frames, *SmProt* small proteins database, *SPENCER* small peptides encoded by non-coding RNAs in cancer patients, *ncEP* ncRNA-encoded peptides, *LncPep* lncRNA-encoded peptides, *FuncPEP* functional ncRNA encoded peptides, *cncRNADB* coding and noncoding RNA database.

### Computational analysis

#### Coding potential assessment

The Coding-Potential Assessment Tool (CPAT) utilizes pure linguistic features calculated from RNA sequences to quickly and accurately assess the likelihood of protein coding, producing probabilities (0 ≤ *p* ≤ 1) based on the input nucleotide sequences or genomic coordinates of RNAs [26]. Another tool, the Coding Potential Calculator (CPC), can estimate a transcript’s protein coding potential by analyzing six sequence features [27]. CPC2, an updated version, boasts a thousand fold increase in speed over its predecessor while enhancing accuracy and maintaining a species-neutral approach [28]. The Coding-Non-Coding Identifying Tool (CNIT) is well-suited for transcriptome analysis, aiding researchers in validating coding or noncoding hypotheses with high accuracy, robustness, and consistency [29]. Phylogenetic Codon Substitution Frequencies (PhyloCSF), developed by the Broad Institute, is a track that assists in identifying functionally conserved, protein-coding regions of genomes [30]. Additionally, COME is a coding potential calculation tool that integrates sequencing-derived or experiment-based features to enhance prediction accuracy and robustness [31]. ORF Finder is a widely used tool for identifying ORFs in lncRNA sequences [32]. Coding Region Identification Tool Invoking Comparative Analysis (CRITICA) includes various programs that search for and rank likely protein-coding ORF sequences [33].

#### Internal ribosomal entry sites (IRESs) analysis

An IRES, or internal ribosome entry site, is an important RNA segment that enables the initiation of translation without relying on the cap structure, playing a key role in protein synthesis [34]. RNA binding proteins (RBPs) can bind to lncRNAs to form ribonucleoprotein (RNP) complexes, which function as well as Kozaks sequence around the AUG start codon to activate translation initiation [35]. Recent studies have highlighted novel functionalities of lncRNAs in IRES elements. Legnini et al., identified that the 5’ UTR of circ-ZNF609 is able to work as an IRES, enabling the encoding of a protein in a splicing-dependent manner [36]. Yu et al., recently identified that DNA damage enhances the interaction of ribosomes with the IRES region of the lncRNA CTBP1-DT. This interaction mitigates negative modulators on the ORF and enhances the translation of the micropeptide DNA damage-upregulated protein (DDUP) through a cap-independent mechanism [37].

#### m^6^A modification prediction

N6-methyladenosine (m^6^A) has been recognized as a prevalent regulatory mechanism that influences RNA expression across various physiological processes [38]. Emerging studies have shown that m^6^A modification accounts for lncRNA translation in mammals [39]. Additionally, different approaches have been developed to predict the m^6^A sites on lncRNAs. DeepM6ASeq, a deep-learning framework, allows for the prediction and visualization of m^6^A sites within sequences [40]. Similarly, SRAMP (sequence-based RNA adenosine methylation site predictor), a web-based tool, offers the capability to identify mammalian m^6^A sites at single-nucleotide resolution [41]. These tools are crucial for advancing our understanding of m^6^A impact on lncRNA function and its broader implications in disease and development.

### Transcriptomic-based method

Over the last decade, researchers have devoted considerable effort to developing high-throughput profiling techniques to analyze the sequences predicted to be translated into ncRNAs, as summarized in Table 1.

Ribosome profiling, also known as Ribo-sequencing or active mRNA translation sequencing (ART-seq), has emerged as a common technique for quantitatively and thoroughly assessing translation. This method involves deep sequencing of ribosome-protected mRNA fragments, allowing researchers to identify hundreds of translated ORFs across various species, including zebrafish and Homo sapiens [42, 43]. Poly-Ribo sequencing is an advanced ribosome profiling technique that leverages active translation and the clustering of multiple ribosomes to minimize false positives [44]. Ribosome-nascent chain complex (RNC) sequencing refers to a technique used to analyze the collection of molecules that comprise a ribosome attached to a nascent polypeptide (protein) during translation [45].

### Proteomics-based method

Researchers have employed proteomics, specifically mass spectrometry (MS), to validate micropeptides encoded by ncRNAs. For instance, Banfai and colleagues conducted a joint analysis of two public datasets that included tandem mass spectrometry (MS/MS) and RNA-seq data from K562 and GM12879 cell lines. Their study examined 79,333 peptides derived from 9,640 lncRNA loci, ultimately identifying 85 unique peptides corresponding to 69 lncRNAs [46]. Additionally, Slavoff utilized a combination of RNA-seq and liquid chromatography-tandem mass spectrometry (LC/MS/MS) methods, and identified 90 small open reading frame-encoded polypeptides (SEPs), 86 of which were characterized in K562 cells [47].

### Experimental identification

Immunoblotting is a straightforward and traditional method used to detect proteins. This technique is particularly valuable for examining the endogenous expression of small peptides. However, the process of creating targeted antibodies presents several challenges. For instance, peptides that contain transmembrane domains may restrict the availability of epitopes suitable for antibody generation [48]. Alternatively, researchers can employ tagging systems, such as GFP-tag or Flag-tag, for validation purposes. These tags are typically cloned into the ORF sequence just before the stop codon, followed by transfection into a cell line. Subsequently, immunoblotting and immunofluorescence (IF) assays are performed to verify the presence of the tagged proteins [49]. Moreover, the CRISPR-Cas9 system offers another approach by facilitating the insertion of a Flag-tag directly before the stop codon of the lncRNA locus within target cells, followed by immunoblotting and IF assays to detection and localization of micropeptide expression [50].

## LncRNA-encoded micropeptides in the immune system and inflammatory response

**Fig. 2:**
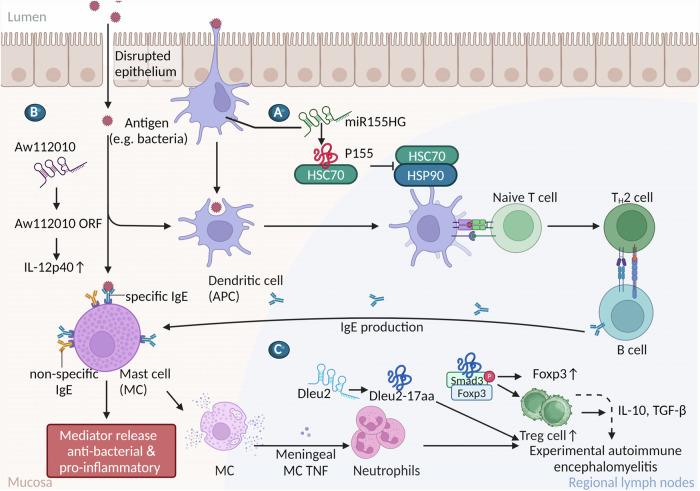
LncRNA-encoded micropeptides in the immune system and inflammatory response. **A** The micropeptide miPEP155 (P155) drives DC-stimulated autoimmune inflammation by disrupting the HSC70-HSP90 machinery. **B** The Aw112010-derived ORF peptide enhances IL-12 signaling. **C** The micropeptide Dleu2-17aa maintains immune homeostasis by interaction with Smad3 and Foxp3.

Recent studies have highlighted the significant role of lncRNA-encoded micropeptides in human innate immunity (Fig. 2). For instance, Niu and colleagues reported that lncRNA miR155HG encodes a 17-aa micropeptide, called miPEP155 (P155). P155 is highly expressed in inflamed antigen-presenting cells and interacts with HSC70 at the adenosine 5′-triphosphate binding domain. It affects the antigen presentation by major histocompatibility complex class II and interferes with the HSC70-HSP90 machinery, thus regulating T-cell priming (Fig. 2A) [51]. Additionally, Jackson et al., reported that a non-canonical ORF peptide derived from Aw112010 exhibits a translational capacity and influences mucosal immunity by enhancing IL-12 stability upon bacterial infection (Fig. 2B) [52]. Tang et al., recently reported that the lncRNA Dleu2-encoded micropeptide Dleu2-17aa can serve as scaffold to promote the interaction between Smad3 and Foxp3, therefore strengthening inducible regulatory T (iTreg) cell generation. Knocking out Dleu2-17aa in mice diminishes the iTreg cell formation and consequently deteriorates experimental autoimmune encephalomyelitis (EAE) (Fig. 2C) [53]. These findings imply the fundamental roles that micropeptides play as modulators of immunological processes.

## LncRNA-encoded micropeptides in mitochondria

**Fig. 3:**
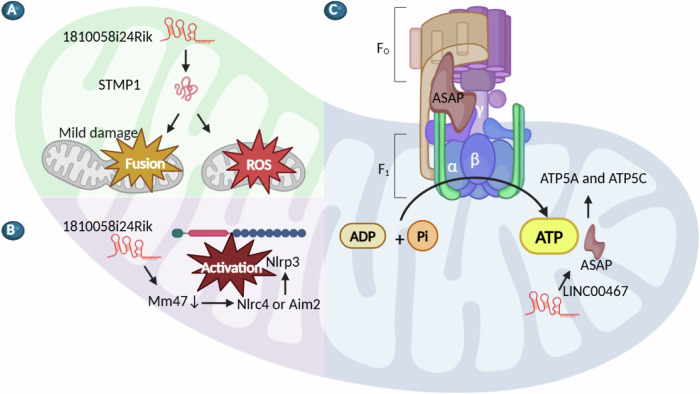
LncRNA-encoded micropeptides in mitochondria. **A** The micropeptide STMP1 enhances mitochondrial fusion and ROS production. **B** The micropeptide Mm47 impacts NIrp3 inflammasome-mediated responses by promoting the interaction between NIrc4 and Aim2. **C** The micropeptide ASAP regulates ATP synthase activity via interaction with ATP5A and ATP5C, eventually affecting colon cancer tumorigenesis in vitro and in vivo.

Mitochondria are dynamic organelles responsible for energy transformation and signaling, crucial for maintaining cellular bioenergetics through ATP production [54]. Recent studies have demonstrated that lncRNA-encoded micropeptides play a crucial role in mitochondrial activity (Table 2). Notably, three different groups have parallelly examined the function of the lncRNA 1810058I24Rik-encoded micropeptide STMP1 in mitochondrial processes. Zheng et al., initially identified STMP1 as a 47-aa mitochondrial micropeptide that is involved in retinal differentiation by promoting the differentiation of bipolar and amacrine cells via the 15-AA N-terminus of STMP1 [55]. They further demonstrated that STMP1 regulates retinal ischemia/reperfusion (IR) via activating microglia, enhancing aerobic glycolysis, and promoting mitochondrial fusion and reactive oxygen species (ROS) production (Fig. 3A) [56]. Xie et al., identified that the inner mitochondrial membrane-located micropeptide STMP1 boosts mitochondrial fission and cell migration by increasing DRP1 expression and facilitating its interaction with MYH9 [57]. Sang et al., characterized STMP1’s promotion of cell cycle arrest by enhancing the activity of mitochondrial complex IV [58]. In addition, Bhatta et al., reported that the lncRNA 1810058I24Rik encoded another micropeptide, called Mm47, which is required for the interaction between Nlrc4 and Aim2, influencing the Nlrp3 inflammasome activity (Fig. 3B) [59]. Moreover, Ge et al., reported that ASAP, a 94-aa micropeptide encoded by lncRNA LINC00467, is involved in mitochondrial metabolism. ASAP regulates ATP synthase activity via interaction with ATP5A and ATP5C, eventually affecting colon cancer tumorigenesis in vitro and in vivo (Fig. 3C) [11].

**Table 2:** LncRNA-encoded micropeptides in mitochondria.

LncRNA	Peptide	Size (aa)	Disease	Function and mechanism	Ref.
1810058I24Rik	STMP1	47	Retinal ischemia/reperfusion injury	Function as a mitochondrion-located micropeptide via influences microglia and inflammasomes.	[56]
LINC00467	ASAP	94	Colorectal cancer	Promote CRC tumorigenesis by regulating ATP synthase and mitochondrial oxygen consumption rate.	[11]
LINC00116	Mtln	56	Respiration and lipid metabolism	Increase mitochondrial membrane potential, respiration rates, and Ca^2+^ retention capacity while decreasing mitochondrial ROS and matrix-free Ca^2+^.	[120]
LINC00116	MOXI	56	Fatty Acid β-Oxidation	Interact with the MTP in the mitochondrial inner membrane and enhances fatty acid oxidation.	[121]
MyolncR4	LEMP	56	Skeletal muscle differentiation	Localize at both the plasma membrane and mitochondria, and associate with multiple mitochondrial proteins.	[86]
LINC00998	SMIM30	59	Liver cancer	SMIM30 localizes in the membranes of the ER and mitochondria and promote the G1/S transition by reducing cytosolic calcium level, thereby enhancing cell proliferation and tumor growth.	[122]
LINC00493	SMIM26	95	Clear cell renal cell carcinoma	Bind to AGK and promotes its localization at mitochondrial, eventually inactivates AKT signaling, and represses cancer metastasis.	[123]
LINC01013	smORF	56	Myocardial fibrosis	smORF localizes in the mitochondrial matrix and leads to myocardial fibrosis.	[124]
LINC00948	MLN	46	Muscle performance	MLN directly interacts with SERCA and impedes Ca^2+^ uptake into the sarcoplasmic reticulum.	[85]
AFAP1-AS1	ATMLP	90	Non-small cell lung cancer	Inhibit NIPSNAP1 transportation and antagonizes the NIPSNAP1-mediated cell autolysosome formation.	[125]

*STMP1* Short transmembrane mitochondrial protein1, *ASAP* ATP synthase-associated peptide, *CRC* Colorectal cancer, *Mtln* Mitoregulin, *MOXI* Micropeptide regulator of β-oxidation, *MTP* mitochondrial trifunctional protein, *LEMP* lncRNA encoded micropeptide, *SMIM30* Small Integral Membrane Protein 30, *SMIM26* Small Integral Membrane Protein 26, *AGK* acylglycerol kinase, *smORF* small Open Reading Frame, *HOXB-AS3* HOXB Cluster Antisense RNA 3, *MLN* myoregulin, *SERCA* sarcoplasmic reticulum Ca2 + -ATPase, *ATMLP* AFAP1-AS1 translated mitochondrial-localized peptide, *NIPSNAP1* non-neuronal SNAP25-like protein homolog 1.

In summary, these studies highlight the fundamental functions of lncRNA-encoded micropeptides in mitochondrial activities.

## LncRNA-encoded micropeptides in cancer

Cancer is the second-leading cause of death worldwide, with approximately 20 million newly-diagnosed cases and approximately 10 million deaths in 2022 [60]. Cancer is a result of the abnormal proliferation of normal cells, through their transformation to tumor cells following a multi-step process that culminates in unconstrained growth, and typically, metastasis. Research shows that micropeptides can influence tumorigenesis via diverse mechanisms (Table 3) [61]. Herein, we will summarize the functions of lncRNA-encoded micropeptides in different cancer types.

**Table 3:** lncRNA-encoded micropeptides in cancer.

Peptide name	Size (aa)	LncRNA	Cancer type	Function and Mechanism	Ref.
HOXB-AS3 peptide	53	HOXB-AS3	Colorectal cancer	Suppress cell growth by inhibition of glucose metabolism reprogramming in colon cancer.	[10]
pep-AP	37	Lnc-AP	Colorectal cancer	Attenuate the pentose phosphate pathway, NADPH/NADP+ and glutathione levels and causing ROS accumulation, sensitize colorectal cancer cells to Oxaliplatin via binding to TALDO1.	[62]
FORCP	79	LINC00675	Colorectal cancer	Inhibit proliferation, clonogenicity and tumorigenesis.	[126]
RBRP	71	LINC00266-1	Colorectal cancer	Bind to m^6^A reader IGF2BP1 and strengthen MYC stability, thereby promoting tumorigenesis.	[63]
ASAP	94	LINC00467	Colorectal cancer	Increase ATP synthase activity and mitochondrial oxygen consumption rate by interaction with ATP5A and ATP5C.	[11]
SRSP	130	LOC90024	Colorectal cancer	Facilitate of the splicing factor SRSF3 binds to exon 3 of Sp4 to produce a long Sp4 isoform, eventually promoting CRC tumorigenesis and progression.	[127]
UBAP1-AST6 peptide	-	UBAP1-AST6	Lung cancer	Enforced UBAP1-AST6 peptide promotes cell growth, whereas UBAP1-AST6 KO inhibits cell proliferation and clone formation.	[64]
DLX6-AS1 ORF	-	DLX6-AS1	Non-small cell lung cancer	Promote cell proliferation, migration and invasion by activating Wnt/β-catenin pathway.	[65]
CASIMO1	10	NR_029453	Breast cancer	Stimulate cell growth by interaction with SQLE and upregulation of ERK phosphorylation.	[68]
PACMP	44	CTD-2256P15.2	Breast cancer	Prevent CtIP from KLHL15-mediated ubiquitination and proteasomal degradation, promote DNA damage-triggered PARylation.	[69]
ASRPS	60	LINC00908	Breast cancer	Decrease of VEGF level via inhibiting STAT3 phosphorylation, eventually repressing tumorigenesis.	[66]
XBP1SBM	21	MLLT4-AS1	Breast cancer	Improve Gln levels, promote angiogenesis and metastasis in TNBC.	[128]
CIP2A-BP	52	LINC00665	Breast cancer	Bind CIP2A to replace PP2A’s B56γ subunit, consequently enhancing PP2A activity and inhibiting PI3K/AKT/NFκB pathway.	[67]
KRASIM	99	NCBP2-AS2	Hepatocellular carcinoma	KRASIM decreases the KRAS protein level, resulting in the inhibition of ERK signaling activity and cell growth and proliferation.	[129]
C20orf204-189AA	189	LINC00176	Hepatocellular carcinoma	Enhance cell proliferation and ribosomal RNA transcription via interaction with nucleolin in HCC.	[71]
SMIM30	59	LINC00998	Hepatocellular carcinoma	Induction of anchoring of SRC/YES1 membrane and activation of MAPK pathway.	[73]
STMP1	47	C7orf73	Hepatocellular carcinoma	Promote mitochondrial fission via increasing dynamin-related protein 1, enhance cell migration via interaction with MYH9; promote the G1/S transition and CCNE2, CDK2, and E2F1 by strengthening mitochondrial complex IV activity.	[57, 58]
AC115619-22aa	22	AC115619	Hepatocellular carcinoma	Repress HCC progression via the interaction with WTAP and impedes the assembly of the m^6^A methyltransferase complex.	[75]
JunBP	174	LINC02551	Hepatocellular carcinoma	JunBP increases phosphorylation of c-Jun and enhances SMAD3 expression.	[130]
PINT87aa	87	LINC-PINT	Hepatocellular carcinoma	PINT87aa induces cell cycle arrest and cellular senescence by directly binding to FOXM1.	[131]
PINT87aa	87	LINC-PINT	Glioblastoma	PINT87aa directly interacts with PAF1c and inhibits the transcriptional elongation of multiple oncogenes.	[132]
MP31	31	PTEN uORF	Glioblastoma	MP31 inhibits lysosome function and blocks lysosome fusion with mitophagosomes by competing with V-ATPase A1.	[79]
APPLE	90	AHS1L-AS1	Acute myeloid leukemia	Promote PABPC1-eIF4G interaction, mRNA looping and enhance translation via interaction with eIF4F.	[76]
YY1BM	21	LINC00278	Esophageal squamous cell carcinoma	Inhibit the interaction between YY1 and AR, thereby decreasing expression of eEF2K through the AR pathway.	[39]
MIAC	51	AC025154.2	Renal cell carcinoma	Inhibit the proliferation and migration capacity by binding to AQP2 protein and inhibiting EREG/EGFR expression.	[77]
TINCR	120	Tincr	Squamous cell carcinoma	Low expressed in SCC and enforced TINCR represses cell and tumor growth.	[78]
NOBODY	71	LINC01420	Cancer-related	NoBody localizes to P-bodies and interacts with mRNA decapping proteins to participate in mRNA turnover and nonsense-mediated decay, and influences nasopharyngeal carcinoma invasion and metastasis.	[133, 134]
MELOE-1	46	Meloe	Melanoma	MELOE1 is involved in tumor-infiltrating lymphocyte.	[135]

*m*^*6*^*A* N^6^-methyladenosine, *ORF* Open reading frame, *HOXB-AS3* HOXB cluster antisense RNA 3, *FORCP* FOXA1-Regulated Conserved Small Protein, *RBRP* RNA-binding regulatory peptide, *ASAP* ATP synthase-associated peptide, *SRSP* serine- and arginine-rich splicing factor 3, *DLX6-AS1* distal-less homeobox 6 antisense 1, *CASIMO1* Cancer-Associated Small Integral Membrane Open reading frame 1, *PACMP* PAR-amplifying and CtIP-maintaining micropeptide, *ASRPS* a small regulatory peptide of STAT3, *XBP1SBM* XBP1s binding micropeptide, *CIP2A-BP* CIP2A binding peptide, *KRASIM* KRAS interaction micropeptide, *SMIM30* small integral membrane protein 30, *STMP1* short transmembrane protein 1, *JunBP* Jun binding micropeptide, *PINT* p53-induced transcript, *PAF1c* polymerase associated factor complex, *APPLE* a peptide located in ER, *YY1BM* Yin Yang 1-binding micropeptide, *AR* androgen receptor, *MIAC* micropeptide inhibiting actin cytoskeleton, *TINCR* Terminal differentiation-Induced Non-Coding RNA, *MP31* a micropeptide encoded by the uORF of PTEN, *uORF* upstream open reading frame, *PTEN* phosphatase and tensin homolog, *NOBODY* non-annotated P-body dissociating polypeptide, *MELOE* melanoma-overexpressed antigen.

### Colon cancer

**Fig. 4:**
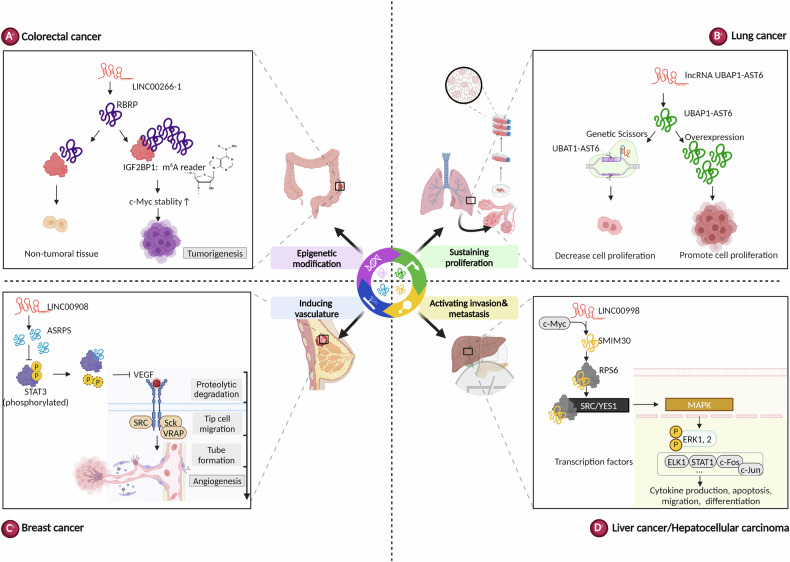
The functions of lncRNA-encoded micropeptides in cancer. **A** The micropeptide RBRP interacts with m^6^A reader IGF2BP1 and strengthens MYC stability in colorectal cancer. **B** Overexpression of micropeptide UBAP1-AST6 promotes cell growth, whereas UBAP1-AST6 KO inhibits cell proliferation in lung cancer. **C** The LINC00908-encoded micropeptide ASRPS inhibits angiogenesis by preventing phosphorylation of STAT3 in breast cancer. **D** The Micropeptide SMIM30 activates MAPK signaling and HCC progression by interacting with the non-receptor tyrosine kinase SRC/YES1.

Huang and colleagues found a reduction of lncRNA HOXB-AS3 in colorectal cancer (CRC) tissues compared to the adjacent non-tumoral colon tissues. Highly metastatic colon cell lines also exhibited a reduction of HOXB-AS3. They found that the lncRNA HOXB-AS3 encodes a conserved 53-aa peptide, and showed that the HOXB-AS3 peptide, but not the lncRNA HOXB-AS3 itself, suppresses CRC growth. Mechanistically, the HOXB-AS3 peptide interacts with the hnRNP A1 protein via an RNA-binding RGG box (RGG) and suppresses hnRNP A1-dependent PKM splicing and miR-18a processing. This interaction prevents hnRNP A1 from binding to flanking PKM E9, effectively antagonizing CRC growth and migration/invasion [10]. In another study, lncRNA AP002387.2 (lnc-AP) is downregulated in chemotherapy-resistant CRC cells, whereas enforced lnc-AP is associated with beneficial clinical outcomes. The authors further found that lnc-AP encodes a micropeptide called pep-AP. Pep-AP and its binding protein TALDO1 co-repress the pentose phosphate pathway (PPP), reducing NADPH/NADP+ and glutathione (GSH) levels. This leads to ROS accumulation and apoptosis, sensitizing CRC cells to oxaliplatin treatment [62]. Additionally, Zhu et al., recently deciphered that the lncRNA LINC00266-1 encodes a 71-amino acid peptide, called RNA-binding regulatory peptide (RBRP) due to its interaction with several functional RNA-binding proteins. RBRP, which is highly expressed in metastatic cell lines and CRC tumors, interacts with the RNA m^6^A reader IGF2BP1 to enhance its recognition of the transcriptional factor MYC, thereby promoting MYC stability (Fig. 4A) [63].

### Lung cancer

Lu et al., reported that the lncRNA-derived micropeptide UBAP1-AST6, is localized in the nucleoli and highly expressed in the lung cancer cell line A549. Overexpression of UBAP1-AST6 promotes cell growth, whereas UBAP1-AST6 KO via CRISPR-Cas9 significantly inhibits cell proliferation and clone formation. However, this overexpression of UBAP1-AST6 is reversed by mutating the start codon ATG, suggesting the coding potential and importance of UBAP1-AST6 in lung cancer (Fig. 4B) [64]. Meanwhile, another lncRNA-encoded peptide called DLX6-AS1 ORF can promote cell proliferation, migration, and invasion by activating the Wnt/β-catenin pathway in non-small cell lung cancer (NSCLC) [65].

### Breast cancer

Wang et al., recently found that the lncRNA LINC00908 encodes a 60-aa micropeptide named ASRPS in triple-negative breast cancer (TNBC). ASRPS is low-expressed in TNBC, and its reduction correlates with poor survival and promotes tumor growth. Functionally, ASRPS interacts with STAT3 and prevents STAT3 phosphorylation and VEGF activation, subsequently repressing tumorigenesis (Fig. 4C) [66]. Another study identified that the lncRNA LINC00665 encodes a micropeptide called CIP2A-BP, which inhibits migration and invasion in breast cancer. The translation of CIP2A-BP is blocked by TGF-β-induced SMAD activation, which promotes the translation inhibitory factor 4E-BP1 and suppresses the initiation factor eIF4E. CIP2A-BP specifically competes with the PP2A subunit B56γ to bind CIP2A, reducing CIP2A/PP2A-mediated activation of the PI3K/AKT/NFκB pathway and thus inhibiting TNBC tumorigenesis [67]. Additionally, the CASMIMO1 peptide, a 10-amino acid microprotein generally located in endosomes, has been shown to play a crucial role in cell lipid homeostasis and breast cancer proliferation. CASIMO1 interacts with squalene epoxidase (SQLE), enhancing SQLE accumulation and ERK phosphorylation, leading to G0/G1 arrest [68]. Recently, a study proposed that lncRNA CTD-2256P15.2 contributes to epirubicin (EPI)-resistant breast tumors. They further found that the lncRNA CTD-2256P15.2 encodes a micropeptide called PAR-amplifying and CtIP-maintaining micropeptide (PACMP), which modulates DNA double-strand break (DSB), chemoresistance, and CtIP protein abundance through KLHL15-mediated degradation. PACMP enhances poly (ADP-ribosyl)ation by PARP1 through its binding to DNA damage-generated poly (ADP-ribose) chains. Targeting PACMP could sensitize tumor cells to various treatments including PARP, ATR, and CDK4/6 inhibitors, ionizing radiation, and camptothecin, opening new avenues for therapeutic strategies to improve clinical outcomes [69].

### Liver cancer

Xu et al., identified a conserved microprotein KRASIM encoded by the lncRNA NCBP2-AS2 by utilizing ribosome profiling in hepatocellular carcinoma (HCC) cells. They noted that KRASIM is expressed at lower levels in HCC compared to normal hepatocytes and found that it inhibits HCC cell growth and proliferation by reducing KRAS protein levels and dampening ERK signaling pathway activity [70]. In other studies, De Lara and Polenkowski identified two lncRNA-encoded peptides, C20orf204-189AA and linc013026-68AA, which correlate with tumor differentiation grade and patient survival. These findings suggest their roles as cancer-specific fine tuners, offering potential targets for therapy in HCC [71, 72]. Using an antibody against ribosomal protein S6 (RPS6), Pang performed a RIP-seq assay and observed that the lncRNA LINC00998 encodes a micropeptide called SMIM30. SMIM30 is induced by MYC and can activate MAPK signaling and HCC progression by interacting with the non-receptor tyrosine kinase SRC/YES1 (Fig. 4D) [73]. Zhang identified that the TGF-β-induced lncRNA LINC02551 encodes a 174-amino-acid peptide, called Jun binding micropeptide (JunBP). JunBP binds c-JUN, enhancing its phosphorylation and affinity for SMAD3, which induces LINC02551 and forms a positive regulatory feedback loop promoting HCC metastasis [74]. Hypoxia-responsive lncRNA AC115619 encodes a micropeptide, AC115619-22aa, in HCC. AC115619-22aa represses HCC progression via the interaction with WTAP and impedes the assembly of the m^6^A methyltransferase complex, therefore affecting the expression of tumor genes including SOCS2 and ATG14 [75].

### Others

Sun and colleagues have recently shown that the micropeptide APPLE, encoded by the lncRNA ASH1L-AS1, is upregulated in Acute Myeloid Leukemia (AML) and associated with poor outcomes in hematopoietic malignancies. Mechanistically, APPLE acts as a novel member of the PABPC1 complex, facilitating the interaction between PABPC1 and eIF4G. This interaction promotes mRNA circularization and eIF4F translation initiation by binding the RRM1 and RRM3 domains of PABPC1, thereby contributing to AML progression [76]. In esophageal squamous cell carcinoma (ESCC), the Y-linked lncRNA LINC00278 encodes a Yin Yang 1 (YY1)-binding micropeptide, designated YY1BM, which inhibits the interaction between YY1 and androgen receptor (AR). This decreases eEF2K expression and promotes cell apoptosis [39]. In renal cell carcinoma (RCC), overexpressed micropeptide MIAC significantly reduces the capacity of cells to proliferate and migrate by binding to AQP2 and reducing EREG/EGFR expression in vitro and in vivo [77]. Furthermore, the terminal differentiation-induced Non-Coding RNA (TINCR) encodes a highly conserved ubiquitin-like microprotein that serves as a tumor suppressor to repress tumor growth of squamous cell carcinoma [78]. In glioblastoma (GBM), the tumor-suppressing micropeptide MP31 disrupts mitochondrial quality control, causing defective mitochondria to accumulate in cells, which in turn results in ROS production and DNA damage [79].

In summary, these novel investigations reveal that the lncRNA-encoded peptides are closely involved in tumor-relevant activities and might become promising targets for cancer treatment.

## LncRNA-encoded micropeptides in other diseases

### Pulmonary hypertension

Increasing studies have shown that micropeptides also participate in the pathogenesis of other diseases. Pulmonary hypertension, characterized by pulmonary blood vessel abnormalities, has been linked to micropeptide involvement. Recently, the Zhu lab reported that lncRNA RPS4L encodes a micropeptide called 40S ribosomal protein S4 X isoform-like (RPS4XL), which promotes pulmonary artery smooth muscle cells (PASMCs) proliferation under hypoxic conditions. RPS4XL binds to RPS6 to inhibit its phosphorylation at Ser240 and Ser244 sites [80]. Additionally, RPS4XL suppresses hypoxia-induced pyroptosis in PASMCs by interacting with the glycosylation site of HSC70 [81]. These findings suggest that RPS4XL could be a potential target for treating pulmonary hypertension.

### Myocardial infarction

Myocardial infarction (MI), or heart attack, occurs when the myocardium receives decreased, or no, blood flow leading to tissue damage or death [82]. Spiroski et al., reported that the lncRNA LINC00961-encoded micropeptide SPAAR, short for small regulatory polypeptide of amino acid response, is expressed mostly in human cardiac endothelial cells and fibroblasts. SPAAR is implicated with fibroblast function, hypoxic response and basal cardiovascular function in adulthood [83]. In a parallel study, Yan and colleagues observed that three micropeptides encoded by lncRNAs are involved in the process of oxidative phosphorylation, and the signaling pathways of calcium and MAPK, thereby regulating cardiomyocyte hypertrophy [84].

### Muscle development

Anderson et al., identified a conserved micropeptide, myoregulin (MLN), coded by a muscle-specific lncRNA. MLN is structurally similar to the membrane pump SERCA inhibitors phospholamban and sarcolipin, therefore inhibiting SERCA by regulating Ca^2+^ uptake into the sarcoplasmic reticulum (SR) [85]. These findings underscore the importance of exploring lncRNA-encoded micropeptides and highlight the complexity of molecular mechanisms underlying disease processes. LncRNA MyolncR4 has been found to encode a 56-aa micropeptide called lncRNA-encoded micropeptide (LEMP). LEMP is a highly conserved peptide among different species and is associated with myogenic differentiation. Mice with LEMP KO using CRISPR-Cas9 exhibit a deficit in muscle formation and development [86]. Nelson et al., addressed a putative muscle-specific lncRNA that encodes a peptide of 34-aa, called dwarf open reading frame (DWORF). Upregulated DWORF promotes peak Ca^2+^ transient amplitude and sarcoplasmic reticulum Ca^2+^ load and enhances SERCA activity in cardiomyocytes of mice [87].

In summary, these findings underscore the importance of exploring lncRNA-encoded micropeptides and highlight the complexity of molecular mechanisms underlying disease processes.

## Conclusions and perspectives

Current research has been intensively exploring the biological roles of lncRNAs. Unlike protein-coding mRNAs, lncRNAs contribute uniquely to several cellular mechanisms such as histone modification, DNA methylation, and transcription regulation [88]. Employing strategies that combine in silico prediction, experimental validation, and functional analysis are essential to better understand the complex operations of biological systems and their evolutionary developments. Moreover, the development of new technologies, including functional proteomics, gene editing, and extensive sequencing methods, has substantially enhanced research into micropeptides encoded by lncRNAs.

Functional studies of micropeptides have uncovered their essential biological functions, including immune system response and mitochondrial metabolism. Increasing studies also demonstrate that micropeptides are involved in the development of human diseases. For example, LINC00665 is upregulated in liver cancer, particularly in the pathological stages III and IV compared to the normal counterparts. The LINC00665-encoded peptide CIP2A-BP-52 competes with PP2A to bind to CIP2A, leading to the release and downregulation of the PI3K/AKT/NFκB pathway, thus silencing invasion and metastasis in liver cancer [89]. This review focuses on the role of micropeptides across cancer types, raising the possibility of their implication as biomarkers or novel therapeutics targets.

Despite significant efforts, there is still a vast challenge to be accomplished in understanding the biological roles of micropeptides. Given their relatively short length, it is crucial to develop specific and effective antibodies for further experimental analysis and clinical inspection. Additionally, considering the cell-specific and tissue-specific phenotypes of lncRNAs, it is vital to determine the level and distribution of micropeptides across tissues. Third but not least, bi-functional lncRNAs, either as coding peptides or ncRNA molecules, require and merit further investigation. A more in-depth study of lncRNAs and their encoded micropeptides will significantly advance research in the life sciences, providing new insights and strategies for cancer therapy in particular.

## References

[1] Crick F. Central dogma of molecular biology. Nature. 1970;227:561–3. 4913914 10.1038/227561a0

[2] Wang KC, Chang HY. Molecular mechanisms of long noncoding RNAs. Mol Cell. 2011;43:904–14. 21925379 10.1016/j.molcel.2011.08.018 PMC3199020

[3] Goodall GJ, Wickramasinghe VO. RNA in cancer. Nat Rev Cancer. 2021;21:22–36. 33082563 10.1038/s41568-020-00306-0

[4] Slack FJ, Chinnaiyan AM. The Role of Non-coding RNAs in Oncology. Cell. 2019;179:1033–55. 31730848 10.1016/j.cell.2019.10.017 PMC7347159

[5] Esteller M. Non-coding RNAs in human disease. Nat Rev Genet. 2011;12:861–74. 22094949 10.1038/nrg3074

[6] Mercer TR, Dinger ME, Mattick JS. Long non-coding RNAs: insights into functions. Nat Rev Genet. 2009;10:155–9. 19188922 10.1038/nrg2521

[7] Ransohoff JD, Wei Y, Khavari PA. The functions and unique features of long intergenic non-coding RNA. Nat Rev Mol Cell Biol. 2018;19:143–57. 29138516 10.1038/nrm.2017.104 PMC5889127

[8] McCabe EM, Rasmussen TP. lncRNA involvement in cancer stem cell function and epithelial-mesenchymal transitions. Semin Cancer Biol. 2021;75:38–48. 33346133 10.1016/j.semcancer.2020.12.012

[9] Wu P, Mo Y, Peng M, Tang T, Zhong Y, Deng X, et al. Emerging role of tumor-related functional peptides encoded by lncRNA and circRNA. Mol Cancer. 2020;19:22. 32019587 10.1186/s12943-020-1147-3 PMC6998289

[10] Huang JZ, Chen M, Chen D, Gao XC, Zhu S, Huang H, et al. A Peptide Encoded by a Putative lncRNA HOXB-AS3 Suppresses Colon Cancer Growth. Mol Cell. 2017;68:171–84.e6. 28985503 10.1016/j.molcel.2017.09.015

[11] Ge Q, Jia D, Cen D, Qi Y, Shi C, Li J, et al. Micropeptide ASAP encoded by LINC00467 promotes colorectal cancer progression by directly modulating ATP synthase activity. J Clin Investig. 2021;131:e152911. 10.1172/JCI152911 PMC8592539 34591791

[12] Statello L, Guo CJ, Chen LL, Huarte M. Gene regulation by long non-coding RNAs and its biological functions. Nat Rev Mol Cell Biol. 2021;22:96–118. 33353982 10.1038/s41580-020-00315-9 PMC7754182

[13] Engreitz JM, Pandya-Jones A, McDonel P, Shishkin A, Sirokman K, Surka C, et al. The Xist lncRNA exploits three-dimensional genome architecture to spread across the X chromosome. Science. 2013;341:1237973. 23828888 10.1126/science.1237973 PMC3778663

[14] Dai T, Zhang X, Zhou X, Hu X, Huang X, Xing F, et al. Long non-coding RNA VAL facilitates PKM2 enzymatic activity to promote glycolysis and malignancy of gastric cancer. Clin Transl Med. 2022;12:e1088. 36229913 10.1002/ctm2.1088 PMC9561166

[15] Yamazaki T, Souquere S, Chujo T, Kobelke S, Chong YS, Fox AH, et al. Functional Domains of NEAT1 Architectural lncRNA Induce Paraspeckle Assembly through Phase Separation. Mol Cell. 2018;70:1038–53.e7. 29932899 10.1016/j.molcel.2018.05.019

[16] Wu M, Xu G, Han C, Luan PF, Xing YH, Nan F, et al. lncRNA SLERT controls phase separation of FC/DFCs to facilitate Pol I transcription. Science. 2021;373:547–55. 34326237 10.1126/science.abf6582

[17] Xing YH, Yao RW, Zhang Y, Guo CJ, Jiang S, Xu G, et al. SLERT Regulates DDX21 Rings Associated with Pol I Transcription. Cell. 2017;169:664–78.e16. 28475895 10.1016/j.cell.2017.04.011

[18] Schlackow M, Nojima T, Gomes T, Dhir A, Carmo-Fonseca M, Proudfoot NJ. Distinctive Patterns of Transcription and RNA Processing for Human lincRNAs. Mol Cell. 2017;65:25–38. 28017589 10.1016/j.molcel.2016.11.029 PMC5222723

[19] Kopp F, Mendell JT. Functional Classification and Experimental Dissection of Long Noncoding RNAs. Cell. 2018;172:393–407. 29373828 10.1016/j.cell.2018.01.011 PMC5978744

[20] Wang X, Zhang X, Dang Y, Li D, Lu G, Chan WY, et al. Long noncoding RNA HCP5 participates in premature ovarian insufficiency by transcriptionally regulating MSH5 and DNA damage repair via YB1. Nucleic Acids Res. 2020;48:4480–91. 32112110 10.1093/nar/gkaa127 PMC7192606

[21] Zhou HZ, Li F, Cheng ST, Xu Y, Deng HJ, Gu DY, et al. DDX17-regulated alternative splicing that produced an oncogenic isoform of PXN-AS1 to promote HCC metastasis. Hepatology. 2022;75:847–65. 34626132 10.1002/hep.32195 PMC9304246

[22] Quinn JJ, Chang HY. Unique features of long non-coding RNA biogenesis and function. Nat Rev Genet. 2016;17:47–62. 26666209 10.1038/nrg.2015.10

[23] Dhir A, Dhir S, Proudfoot NJ, Jopling CL. Microprocessor mediates transcriptional termination of long noncoding RNA transcripts hosting microRNAs. Nat Struct Mol Biol. 2015;22:319–27. 25730776 10.1038/nsmb.2982 PMC4492989

[24] Rohrig H, Schmidt J, Miklashevichs E, Schell J, John M. Soybean ENOD40 encodes two peptides that bind to sucrose synthase. Proc Natl Acad Sci USA. 2002;99:1915–20. 11842184 10.1073/pnas.022664799 PMC122294

[25] Gultyaev AP, Koster C, van Batenburg DC, Sistermans T, van Belle N, Vijfvinkel D, et al. Conserved structured domains in plant non-coding RNA enod40, their evolution and recruitment of sequences from transposable elements. NAR Genom Bioinform. 2023;5:091. 10.1093/nargab/lqad091 PMC10578108 37850034

[26] Wang L, Park HJ, Dasari S, Wang S, Kocher JP, Li W. CPAT: Coding-Potential Assessment Tool using an alignment-free logistic regression model. Nucleic Acids Res. 2013;41:e74. 23335781 10.1093/nar/gkt006 PMC3616698

[27] Kong L, Zhang Y, Ye ZQ, Liu XQ, Zhao SQ, Wei L, et al. CPC: assess the protein-coding potential of transcripts using sequence features and support vector machine. Nucleic Acids Res. 2007;35:W345–9. 17631615 10.1093/nar/gkm391 PMC1933232

[28] Kang YJ, Yang DC, Kong L, Hou M, Meng YQ, Wei L, et al. CPC2: a fast and accurate coding potential calculator based on sequence intrinsic features. Nucleic Acids Res. 2017;45:W12–W6. 28521017 10.1093/nar/gkx428 PMC5793834

[29] Guo JC, Fang SS, Wu Y, Zhang JH, Chen Y, Liu J, et al. CNIT: a fast and accurate web tool for identifying protein-coding and long non-coding transcripts based on intrinsic sequence composition. Nucleic Acids Res. 2019;47:W516–W22. 31147700 10.1093/nar/gkz400 PMC6602462

[30] Mudge JM, Jungreis I, Hunt T, Gonzalez JM, Wright JC, Kay M, et al. Discovery of high-confidence human protein-coding genes and exons by whole-genome PhyloCSF helps elucidate 118 GWAS loci. Genome Res. 2019;29:2073–87. 31537640 10.1101/gr.246462.118 PMC6886504

[31] Hu L, Xu Z, Hu B, Lu ZJ. COME: a robust coding potential calculation tool for lncRNA identification and characterization based on multiple features. Nucleic Acids Res. 2017;45:e2. 27608726 10.1093/nar/gkw798 PMC5224497

[32] Sayers EW, Beck J, Bolton EE, Bourexis D, Brister JR, Canese K, et al. Database resources of the National Center for Biotechnology Information. Nucleic Acids Res. 2021;49:D10–D7. 33095870 10.1093/nar/gkaa892 PMC7778943

[33] Badger JH, Olsen GJ. CRITICA: coding region identification tool invoking comparative analysis. Mol Biol Evol. 1999;16:512–24. 10331277 10.1093/oxfordjournals.molbev.a026133

[34] Molla A, Jang SK, Paul AV, Reuer Q, Wimmer E. Cardioviral internal ribosomal entry site is functional in a genetically engineered dicistronic poliovirus. Nature. 1992;356:255–7. 1313153 10.1038/356255a0

[35] Leppek K, Das R, Barna M. Functional 5’ UTR mRNA structures in eukaryotic translation regulation and how to find them. Nat Rev Mol Cell Biol. 2018;19:158–74. 29165424 10.1038/nrm.2017.103 PMC5820134

[36] Legnini I, Di Timoteo G, Rossi F, Morlando M, Briganti F, Sthandier O, et al. Circ-ZNF609 Is a Circular RNA that Can Be Translated and Functions in Myogenesis. Mol Cell. 2017;66:22–37.e9. 28344082 10.1016/j.molcel.2017.02.017 PMC5387670

[37] Yu R, Hu Y, Zhang S, Li X, Tang M, Yang M, et al. LncRNA CTBP1-DT-encoded microprotein DDUP sustains DNA damage response signalling to trigger dual DNA repair mechanisms. Nucleic Acids Res. 2022;50:8060–79. 35849344 10.1093/nar/gkac611 PMC9371908

[38] Zaccara S, Ries RJ, Jaffrey SR. Reading, writing and erasing mRNA methylation. Nat Rev Mol Cell Biol. 2019;20:608–24. 31520073 10.1038/s41580-019-0168-5

[39] Wu S, Zhang L, Deng J, Guo B, Li F, Wang Y, et al. A Novel Micropeptide Encoded by Y-Linked LINC00278 Links Cigarette Smoking and AR Signaling in Male Esophageal Squamous Cell Carcinoma. Cancer Res. 2020;80:2790–803. 32169859 10.1158/0008-5472.CAN-19-3440

[40] Zhang Y, Hamada M. DeepM6ASeq: prediction and characterization of m6A-containing sequences using deep learning. BMC Bioinforma. 2018;19:524. 10.1186/s12859-018-2516-4 PMC6311933 30598068

[41] Zhou Y, Zeng P, Li YH, Zhang Z, Cui Q. SRAMP: prediction of mammalian N6-methyladenosine (m6A) sites based on sequence-derived features. Nucleic Acids Res. 2016;44:e91. 26896799 10.1093/nar/gkw104 PMC4889921

[42] Calviello L, Mukherjee N, Wyler E, Zauber H, Hirsekorn A, Selbach M, et al. Detecting actively translated open reading frames in ribosome profiling data. Nat Methods. 2016;13:165–70. 26657557 10.1038/nmeth.3688

[43] Bazzini AA, Johnstone TG, Christiano R, Mackowiak SD, Obermayer B, Fleming ES, et al. Identification of small ORFs in vertebrates using ribosome footprinting and evolutionary conservation. EMBO J. 2014;33:981–93. 24705786 10.1002/embj.201488411 PMC4193932

[44] Aspden JL, Eyre-Walker YC, Phillips RJ, Amin U, Mumtaz MA, Brocard M, et al. Extensive translation of small Open Reading Frames revealed by Poly-Ribo-Seq. Elife. 2014;3:e03528. 25144939 10.7554/eLife.03528 PMC4359375

[45] Zhao J, Qin B, Nikolay R, Spahn CMT, Zhang G Translatomics: The Global View of Translation. Int J Mol Sci. 2019;20:212. 10.3390/ijms20010212 PMC6337585 30626072

[46] Bánfai B, Jia H, Khatun J, Wood E, Risk B, Gundling WE Jr, et al. Long noncoding RNAs are rarely translated in two human cell lines. Genome Res. 2012;22:1646–57. 22955977 10.1101/gr.134767.111 PMC3431482

[47] Slavoff SA, Mitchell AJ, Schwaid AG, Cabili MN, Ma J, Levin JZ, et al. Peptidomic discovery of short open reading frame-encoded peptides in human cells. Nat Chem Biol. 2013;9:59–64. 23160002 10.1038/nchembio.1120 PMC3625679

[48] Li J, Qu L, Sang L, Wu X, Jiang A, Liu J, et al. Micropeptides translated from putative long non-coding RNAs. Acta Biochim Biophys Sin (Shanghai). 2022;54:292–300. 35538037 10.3724/abbs.2022010 PMC9827906

[49] Pan J, Wang R, Shang F, Ma R, Rong Y, Zhang Y. Functional Micropeptides Encoded by Long Non-Coding RNAs: A Comprehensive Review. Front Mol Biosci. 2022;9:817517. 35769907 10.3389/fmolb.2022.817517 PMC9234465

[50] Yeasmin F, Yada T, Akimitsu N. Micropeptides Encoded in Transcripts Previously Identified as Long Noncoding RNAs: A New Chapter in Transcriptomics and Proteomics. Front Genet. 2018;9:144. 29922328 10.3389/fgene.2018.00144 PMC5996887

[51] Niu L, Lou F, Sun Y, Sun L, Cai X, Liu Z, et al. A micropeptide encoded by lncRNA MIR155HG suppresses autoimmune inflammation via modulating antigen presentation. Sci Adv. 2020;6:eaaz2059. 32671205 10.1126/sciadv.aaz2059 PMC7314557

[52] Jackson R, Kroehling L, Khitun A, Bailis W, Jarret A, York AG, et al. The translation of non-canonical open reading frames controls mucosal immunity. Nature. 2018;564:434–8. 30542152 10.1038/s41586-018-0794-7 PMC6939389

[53] Tang S, Zhang J, Lou F, Zhou H, Cai X, Wang Z, et al. A lncRNA Dleu2-encoded peptide relieves autoimmunity by facilitating Smad3-mediated Treg induction. EMBO Rep. 2024;25:1208–32. 38291338 10.1038/s44319-024-00070-4 PMC10933344

[54] Picard M, Shirihai OS. Mitochondrial signal transduction. Cell Metab. 2022;34:1620–53. 36323233 10.1016/j.cmet.2022.10.008 PMC9692202

[55] Zheng X, Guo Y, Zhang R, Chen H, Liu S, Qiu S, et al. The mitochondrial micropeptide Stmp1 promotes retinal cell differentiation. Biochem Biophys Res Commun. 2022;636:79–86. 36368158 10.1016/j.bbrc.2022.10.107

[56] Zheng X, Wang M, Liu S, Chen H, Li Y, Yuan F, et al. A lncRNA-encoded mitochondrial micropeptide exacerbates microglia-mediated neuroinflammation in retinal ischemia/reperfusion injury. Cell Death Dis. 2023;14:126. 36792584 10.1038/s41419-023-05617-2 PMC9932084

[57] Xie C, Wang FY, Sang Y, Chen B, Huang JH, He FJ, et al. Mitochondrial Micropeptide STMP1 Enhances Mitochondrial Fission to Promote Tumor Metastasis. Cancer Res. 2022;82:2431–43. 35544764 10.1158/0008-5472.CAN-21-3910

[58] Sang Y, Liu JY, Wang FY, Luo XY, Chen ZQ, Zhuang SM, et al. Mitochondrial micropeptide STMP1 promotes G1/S transition by enhancing mitochondrial complex IV activity. Mol Ther. 2022;30:2844–55. 35450818 10.1016/j.ymthe.2022.04.012 PMC9372290

[59] Bhatta A, Atianand M, Jiang Z, Crabtree J, Blin J, Fitzgerald KA. A Mitochondrial Micropeptide Is Required for Activation of the Nlrp3 Inflammasome. J Immunol. 2020;204:428–37. 31836654 10.4049/jimmunol.1900791 PMC7370245

[60] Bray F, Laversanne M, Sung H, Ferlay J, Siegel RL, Soerjomataram I, et al. Global cancer statistics 2022: GLOBOCAN estimates of incidence and mortality worldwide for 36 cancers in 185 countries. CA Cancer J Clin. 2024;74:229–63. 38572751 10.3322/caac.21834

[61] Ye M, Zhang J, Wei M, Liu B, Dong K. Emerging role of long noncoding RNA-encoded micropeptides in cancer. Cancer Cell Int. 2020;20:506. 33088214 10.1186/s12935-020-01589-x PMC7565808

[62] Wang X, Zhang H, Yin S, Yang Y, Yang H, Yang J, et al. lncRNA-encoded pep-AP attenuates the pentose phosphate pathway and sensitizes colorectal cancer cells to Oxaliplatin. EMBO Rep. 2022;23:e53140. 34779552 10.15252/embr.202153140 PMC8728603

[63] Zhu S, Wang JZ, Chen D, He YT, Meng N, Chen M, et al. An oncopeptide regulates m(6)A recognition by the m(6)A reader IGF2BP1 and tumorigenesis. Nat Commun. 2020;11:1685. 32245947 10.1038/s41467-020-15403-9 PMC7125119

[64] Lu S, Zhang J, Lian X, Sun L, Meng K, Chen Y, et al. A hidden human proteome encoded by ‘non-coding’ genes. Nucleic Acids Res. 2019;47:8111–25. 31340039 10.1093/nar/gkz646 PMC6735797

[65] Xu X, Zhang Y, Wang M, Zhang X, Jiang W, Wu S, et al. A Peptide Encoded by a Long Non-Coding RNA DLX6-AS1 Facilitates Cell Proliferation, Migration, and Invasion by Activating the wnt/beta-Catenin Signaling Pathway in Non-Small-Cell Lung Cancer Cell. Crit Rev Eukaryot Gene Expr. 2022;32:43–53. 36017915 10.1615/CritRevEukaryotGeneExpr.2022043172

[66] Wang Y, Wu S, Zhu X, Zhang L, Deng J, Li F, et al. LncRNA-encoded polypeptide ASRPS inhibits triple-negative breast cancer angiogenesis. J Exp Med. 2020;217:e20190950. 10.1084/jem.20190950 PMC7062514 31816634

[67] Guo B, Wu S, Zhu X, Zhang L, Deng J, Li F, et al. Micropeptide CIP2A-BP encoded by LINC00665 inhibits triple-negative breast cancer progression. EMBO J. 2020;39:e102190. 31755573 10.15252/embj.2019102190 PMC6939193

[68] Polycarpou-Schwarz M, Groß M, Mestdagh P, Schott J, Grund SE, Hildenbrand C, et al. The cancer-associated microprotein CASIMO1 controls cell proliferation and interacts with squalene epoxidase modulating lipid droplet formation. Oncogene. 2018;37:4750–68. 29765154 10.1038/s41388-018-0281-5

[69] Zhang C, Zhou B, Gu F, Liu H, Wu H, Yao F, et al. Micropeptide PACMP inhibition elicits synthetic lethal effects by decreasing CtIP and poly(ADP-ribosyl)ation. Mol Cell. 2022;82:1297–312 e8. 35219381 10.1016/j.molcel.2022.01.020

[70] Xu W, Deng B, Lin P, Liu C, Li B, Huang Q, et al. Ribosome profiling analysis identified a KRAS-interacting microprotein that represses oncogenic signaling in hepatocellular carcinoma cells. Sci China Life Sci. 2020;63:529–42. 31240521 10.1007/s11427-019-9580-5

[71] Burbano De Lara S, Tran DDH, Allister AB, Polenkowski M, Nashan B, Koch M, et al. C20orf204, a hepatocellular carcinoma-specific protein interacts with nucleolin and promotes cell proliferation. Oncogenesis. 2021;10:31. 33731669 10.1038/s41389-021-00320-3 PMC7969625

[72] Polenkowski M, Burbano de Lara S, Allister AB, Nguyen TNQ, Tamura T, Tran DDH Identification of Novel Micropeptides Derived from Hepatocellular Carcinoma-Specific Long Noncoding RNA. Int J Mol Sci. 2021;23:58. 10.3390/ijms23010058 PMC8744898 35008483

[73] Pang Y, Liu Z, Han H, Wang B, Li W, Mao C, et al. Peptide SMIM30 promotes HCC development by inducing SRC/YES1 membrane anchoring and MAPK pathway activation. J Hepatol. 2020;73:1155–69. 32461121 10.1016/j.jhep.2020.05.028

[74] Zhang H, Liao Z, Wang W, Liu Y, Zhu H, Liang H, et al. A micropeptide JunBP regulated by TGF-beta promotes hepatocellular carcinoma metastasis. Oncogene. 2023;42:113–23. 36380240 10.1038/s41388-022-02518-0 PMC9816058

[75] Zhang Q, Wei T, Yan L, Zhu S, Jin W, Bai Y, et al. Hypoxia-Responsive lncRNA AC115619 Encodes a Micropeptide That Suppresses m6A Modifications and Hepatocellular Carcinoma Progression. Cancer Res. 2023;83:2496–512. 37326474 10.1158/0008-5472.CAN-23-0337

[76] Sun L, Wang W, Han C, Huang W, Sun Y, Fang K, et al. The oncomicropeptide APPLE promotes hematopoietic malignancy by enhancing translation initiation. Mol Cell. 2021;81:4493–508.e9. 34555354 10.1016/j.molcel.2021.08.033

[77] Li M, Liu G, Jin X, Guo H, Setrerrahmane S, Xu X, et al. Micropeptide MIAC inhibits the tumor progression by interacting with AQP2 and inhibiting EREG/EGFR signaling in renal cell carcinoma. Mol Cancer. 2022;21:181. 36117171 10.1186/s12943-022-01654-1 PMC9484220

[78] Morgado-Palacin L, Brown JA, Martinez TF, Garcia-Pedrero JM, Forouhar F, Quinn SA, et al. The TINCR ubiquitin-like microprotein is a tumor suppressor in squamous cell carcinoma. Nat Commun. 2023;14:1328. 36899004 10.1038/s41467-023-36713-8 PMC10006087

[79] Huang N, Chen Z, Yang X, Gao Y, Zhong J, Li Y, et al. Upstream open reading frame-encoded MP31 disrupts the mitochondrial quality control process and inhibits tumorigenesis in glioblastoma. Neuro Oncol. 2023;25:1947–62. 37280112 10.1093/neuonc/noad099 PMC10628964

[80] Li Y, Zhang J, Sun H, Chen Y, Li W, Yu X, et al. lnc-Rps4l-encoded peptide RPS4XL regulates RPS6 phosphorylation and inhibits the proliferation of PASMCs caused by hypoxia. Mol Ther. 2021;29:1411–24. 33429084 10.1016/j.ymthe.2021.01.005 PMC8058491

[81] Li Y, Zhang J, Sun H, Yu X, Chen Y, Ma C, et al. RPS4XL encoded by lnc-Rps4l inhibits hypoxia-induced pyroptosis by binding HSC70 glycosylation site. Mol Ther Nucleic Acids. 2022;28:920–34. 35757299 10.1016/j.omtn.2022.05.033 PMC9185019

[82] Reynolds HR, Smilowitz NR. Myocardial Infarction with Nonobstructive Coronary Arteries. Annu Rev Med. 2023;74:171–88. 36179347 10.1146/annurev-med-042921-111727

[83] Spiroski AM, Sanders R, Meloni M, McCracken IR, Thomson A, Brittan M, et al. The Influence of the LINC00961/SPAAR Locus Loss on Murine Development, Myocardial Dynamics, and Cardiac Response to Myocardial Infarction. Int J Mol Sci. 2021;22:969. 10.3390/ijms22020969 PMC7835744 33478078

[84] Yan Y, Tang R, Li B, Cheng L, Ye S, Yang T, et al. The cardiac translational landscape reveals that micropeptides are new players involved in cardiomyocyte hypertrophy. Mol Ther. 2021;29:2253–67. 33677093 10.1016/j.ymthe.2021.03.004 PMC8261087

[85] Anderson DM, Anderson KM, Chang CL, Makarewich CA, Nelson BR, McAnally JR, et al. A micropeptide encoded by a putative long noncoding RNA regulates muscle performance. Cell. 2015;160:595–606. 25640239 10.1016/j.cell.2015.01.009 PMC4356254

[86] Wang L, Fan J, Han L, Qi H, Wang Y, Wang H, et al. The micropeptide LEMP plays an evolutionarily conserved role in myogenesis. Cell Death Dis. 2020;11:357. 32393776 10.1038/s41419-020-2570-5 PMC7214441

[87] Nelson BR, Makarewich CA, Anderson DM, Winders BR, Troupes CD, Wu F, et al. A peptide encoded by a transcript annotated as long noncoding RNA enhances SERCA activity in muscle. Science. 2016;351:271–5. 26816378 10.1126/science.aad4076 PMC4892890

[88] Herman AB, Tsitsipatis D, Gorospe M. Integrated lncRNA function upon genomic and epigenomic regulation. Mol Cell. 2022;82:2252–66. 35714586 10.1016/j.molcel.2022.05.027 PMC9219586

[89] Bian X, Jiang H, Meng Y, Li YP, Fang J, Lu Z. Regulation of gene expression by glycolytic and gluconeogenic enzymes. Trends Cell Biol. 2022;32:786–99. 35300892 10.1016/j.tcb.2022.02.003

[90] Wheeler DL, Church DM, Federhen S, Lash AE, Madden TL, Pontius JU, et al. Database resources of the National Center for Biotechnology. Nucleic Acids Res. 2003;31:28–33. 12519941 10.1093/nar/gkg033 PMC165480

[91] Hanada K, Akiyama K, Sakurai T, Toyoda T, Shinozaki K, Shiu SH. sORF finder: a program package to identify small open reading frames with high coding potential. Bioinforma (Oxf, Engl). 2010;26:399–400. 10.1093/bioinformatics/btp688 20008477

[92] Varabyou A, Erdogdu B, Salzberg SL, Pertea M. Investigating Open Reading Frames in Known and Novel Transcripts using ORFanage. Nat Comput Science. 2023;3:700–8. 10.1038/s43588-023-00496-1 PMC10718564 38098813

[93] Lin MF, Jungreis I, Kellis M. PhyloCSF: a comparative genomics method to distinguish protein coding and non-coding regions. Bioinformatics. 2011;27:i275–82. 21685081 10.1093/bioinformatics/btr209 PMC3117341

[94] Yang Z. PAML 4: phylogenetic analysis by maximum likelihood. Mol Biol Evol. 2007;24:1586–91. 17483113 10.1093/molbev/msm088

[95] Yang Z, Nielsen R. Estimating synonymous and nonsynonymous substitution rates under realistic evolutionary models. Mol Biol Evol. 2000;17:32–43. 10666704 10.1093/oxfordjournals.molbev.a026236

[96] Arrial RT, Togawa RC, Brigido M.d.M. Screening non-coding RNAs in transcriptomes from neglected species using PORTRAIT: case study of the pathogenic fungus Paracoccidioides brasiliensis. BMC Bioinforma. 2009;10:239 10.1186/1471-2105-10-239 PMC2731755 19653905

[97] Mokrejs M, Masek T, Vopálensky V, Hlubucek P, Delbos P, Pospísek M. IRESite-a tool for the examination of viral and cellular internal ribosome entry sites. Nucleic Acids Res. 2010;38:D131–D136. 19917642 10.1093/nar/gkp981 PMC2808886

[98] Wang J, Gribskov M. IRESpy: an XGBoost model for prediction of internal ribosome entry sites. BMC Bioinforma. 2019;20:409. 10.1186/s12859-019-2999-7 PMC6664791 31362694

[99] Zhao J, Li Y, Wang C, Zhang H, Zhang H, Jiang B, et al. IRESbase: A Comprehensive Database of Experimentally Validated Internal Ribosome Entry Sites. Genom Proteom Bioinform. 2020;18:129–39. 10.1016/j.gpb.2020.03.001 PMC7646085 32512182

[100] Zhao J, Wu J, Xu T, Yang Q, He J, Song X. IRESfinder: Identifying RNA internal ribosome entry site in eukaryotic cell using framed k-mer features. J Genet Genomics = Yi Chuan Xue Bao. 2018;45:403–6. 30054216 10.1016/j.jgg.2018.07.006

[101] Kolekar P, Pataskar A, Kulkarni-Kale U, Pal J, Kulkarni A. IRESPred: Web Server for Prediction of Cellular and Viral Internal Ribosome Entry Site (IRES). Sci Rep. 2016;6:27436. 27264539 10.1038/srep27436 PMC4893748

[102] Chen K, Wei Z, Zhang Q, Wu X, Rong R, Lu Z, et al. WHISTLE: a high-accuracy map of the human N6-methyladenosine (m6A) epitranscriptome predicted using a machine learning approach. Nucleic Acids Res. 2019;47:e41. 30993345 10.1093/nar/gkz074 PMC6468314

[103] Li G-Q, Liu Z, Shen HB, Yu DJ. TargetM6A: Identifying N6-Methyladenosine Sites From RNA Sequences via Position-Specific Nucleotide Propensities and a Support Vector Machine. IEEE Trans Nanobioscience. 2016;15:674–82. 27552763 10.1109/TNB.2016.2599115

[104] Ingolia NT. Ribosome Footprint Profiling of Translation throughout the Genome. Cell. 2016;165:22–33. 27015305 10.1016/j.cell.2016.02.066 PMC4917602

[105] Xiao Z, Huang R, Xing X, Chen Y, Deng H, Yang X. De novo annotation and characterization of the translatome with ribosome profiling data. Nucleic Acids Res. 2018;46:e61. 29538776 10.1093/nar/gky179 PMC6007384

[106] Zhao P, Zhong J, Liu W, Zhao J, Zhang G. Protein-Level Integration Strategy of Multiengine MS Spectra Search Results for Higher Confidence and Sequence Coverage. J Proteome Res. 2017;16:4446–54. 28965417 10.1021/acs.jproteome.7b00463

[107] Dunn JG, Weissman JS. Plastid: nucleotide-resolution analysis of next-generation sequencing and genomics data. BMC Genomics. 2016;17:958. 27875984 10.1186/s12864-016-3278-x PMC5120557

[108] Fields AP, Rodriguez EH, Jovanovic M, Stern-Ginossar N, Haas BJ, Mertins P, et al. A Regression-Based Analysis of Ribosome-Profiling Data Reveals a Conserved Complexity to Mammalian Translation. Mol Cell. 2015;60:816–27. 26638175 10.1016/j.molcel.2015.11.013 PMC4720255

[109] Ingolia NT, Brar GA, Stern-Ginossar N, Harris MS, Talhouarne GJ, Jackson SE, et al. Ribosome profiling reveals pervasive translation outside of annotated protein-coding genes. Cell Rep. 2014;8:1365–79. 25159147 10.1016/j.celrep.2014.07.045 PMC4216110

[110] Mayor-Ruiz C, Dominguez O, Fernandez-Capetillo O. TrapSeq: An RNA Sequencing-Based Pipeline for the Identification of Gene-Trap Insertions in Mammalian Cells. J Mol Biol. 2017;429:2780–9. 28782559 10.1016/j.jmb.2017.07.020 PMC5695663

[111] Olexiouk V, Crappé J, Verbruggen S, Verhegen K, Martens L, Menschaert G. sORFs.org: a repository of small ORFs identified by ribosome profiling. Nucleic Acids Res. 2016;44:D324–D329. 26527729 10.1093/nar/gkv1175 PMC4702841

[112] Li Y, Zhou H, Chen X, Zheng Y, Kang Q, Hao D, et al. SmProt: A Reliable Repository with Comprehensive Annotation of Small Proteins Identified from Ribosome Profiling. Genomics, Proteom Bioinforma. 2021;19:602–10. 10.1016/j.gpb.2021.09.002 PMC9039559 34536568

[113] Leblanc S, Yala F, Provencher N, Lucier JF, Levesque M, Lapointe X, et al. OpenProt 2.0 builds a path to the functional characterization of alternative proteins. Nucleic Acids Res. 2024;52:D522–D528. 37956315 10.1093/nar/gkad1050 PMC10767855

[114] Choteau, SA, et al. MetamORF: a repository of unique short open reading frames identified by both experimental and computational approaches for gene and metagene analyses. Database : the Journal of Biological Databases and Curation, 2021;2021:baab032. 10.1093/database/baab032 PMC8218702 34156446

[115] Luo X, et al. SPENCER: a comprehensive database for small peptides encoded by noncoding RNAs in cancer patients. Nucleic Acids Res. 2022;50:D1373–D1381. 34570216 10.1093/nar/gkab822 PMC8728293

[116] Liu H, Zhou X, Yuan M, Zhou S, Huang YE, Hou F, et al. ncEP: A Manually Curated Database for Experimentally Validated ncRNA-encoded Proteins or Peptides. J Mol Biol. 2020;432:3364–8. 32105730 10.1016/j.jmb.2020.02.022

[117] Liu T, Wu J, Wu Y, Hu W, Fang Z, Wang Z, et al. LncPep: A Resource of Translational Evidences for lncRNAs. Front Cell Developmental Biol. 2022;10:795084. 10.3389/fcell.2022.795084 PMC8819059 35141219

[118] Dragomir, MP, et al. FuncPEP: A Database of Functional Peptides Encoded by Non-Coding RNAs. Non-coding RNA, 2020;6:41. 10.3390/ncrna6040041 PMC7712257 32977531

[119] Huang Y, Wang J, Zhao Y, Wang H, Liu T, Li Y, et al. cncRNAdb: a manually curated resource of experimentally supported RNAs with both protein-coding and noncoding function. Nucleic Acids Res. 2021;49:D65–D70. 33010163 10.1093/nar/gkaa791 PMC7778915

[120] Stein CS, Jadiya P, Zhang X, McLendon JM, Abouassaly GM, Witmer NH, et al. Mitoregulin: A lncRNA-Encoded Microprotein that Supports Mitochondrial Supercomplexes and Respiratory Efficiency. Cell Rep. 2018;23:3710–3720-.e8. 29949756 10.1016/j.celrep.2018.06.002 PMC6091870

[121] Makarewich CA, Baskin KK, Munir AZ, Bezprozvannaya S, Sharma G, Khemtong C, et al. MOXI Is a Mitochondrial Micropeptide That Enhances Fatty Acid β-Oxidation. Cell Rep. 2018;23:3701–9. 29949755 10.1016/j.celrep.2018.05.058 PMC6066340

[122] Yang JE, Zhong WJ, Li JF, Lin YY, Liu FT, Tian H, et al. LINC00998-encoded micropeptide SMIM30 promotes the G1/S transition of cell cycle by regulating cytosolic calcium level. Mol Oncol. 2023;17:901–16. 36495128 10.1002/1878-0261.13358 PMC10158777

[123] Meng, K, et al., LINC00493‐encoded microprotein SMIM26 exerts anti‐metastatic activity in renal cell carcinoma. EMBO rep;2023;24:e56282. 10.15252/embr.202256282 PMC10240204 37009826

[124] Quaife NM, Chothani S, Schulz JF, Lindberg EL, Vanezis K, Adami E, et al. LINC01013 Is a Determinant of Fibroblast Activation and Encodes a Novel Fibroblast-Activating Micropeptide. J Cardiovasc Transl Res. 2023;16:77–85. 35759180 10.1007/s12265-022-10288-z PMC9944705

[125] Pei H, Dai Y, Yu Y, Tang J, Cao Z, Zhang Y, et al. The Tumorigenic Effect of lncRNA AFAP1-AS1 is Mediated by Translated Peptide ATMLP Under the Control of m(6) A Methylation. Adv Sci (Weinh). 2023;10:e2300314. 36871154 10.1002/advs.202300314 PMC10161021

[126] Li, XL, et al. A small protein encoded by a putative lncRNA regulates apoptosis and tumorigenicity in human colorectal cancer cells. ELife, 2020;9. 10.7554/eLife.53734 PMC7673786 33112233

[127] Meng N, Chen M, Chen D, Chen XH, Wang JZ, Zhu S, et al. Small Protein Hidden in lncRNA LOC90024 Promotes “Cancerous” RNA Splicing and Tumorigenesis. Adv Sci (Weinh, Baden -Wurtt, Ger). 2020;7:1903233. 10.1002/advs.201903233 PMC7237858 32440474

[128] Wu S, Guo B, Zhang L, Zhu X, Zhao P, Deng J, et al. A micropeptide XBP1SBM encoded by lncRNA promotes angiogenesis and metastasis of TNBC via XBP1s pathway. Oncogene. 2022;41:2163–72. 35197570 10.1038/s41388-022-02229-6

[129] Xu W, Deng B, Lin P, Liu C, Li B, Huang Q, et al. Ribosome profiling analysis identified a KRAS-interacting microprotein that represses oncogenic signaling in hepatocellular carcinoma cells. Science China. Life Sci. 2020;63:529–42. 10.1007/s11427-019-9580-5 31240521

[130] Kan L, Yang M, Zhang H. Long noncoding RNA PSMA3-AS1 functions as a competing endogenous RNA to promote gastric cancer progression by regulating the miR-329-3p/ALDOA axis. Biol Direct. 2023;18:36. 37403106 10.1186/s13062-023-00392-8 PMC10318671

[131] Xiang X, Fu Y, Zhao K, Miao R, Zhang X, Ma X, et al. Cellular senescence in hepatocellular carcinoma induced by a long non-coding RNA-encoded peptide PINT87aa by blocking FOXM1-mediated PHB2. Theranostics. 2021;11:4929–44. 33754036 10.7150/thno.55672 PMC7978318

[132] Zhang M, Zhao K, Xu X, Yang Y, Yan S, Wei P, et al. A peptide encoded by circular form of LINC-PINT suppresses oncogenic transcriptional elongation in glioblastoma. Nat Commun. 2018;9:4475. 30367041 10.1038/s41467-018-06862-2 PMC6203777

[133] D'lima NG, Ma J, Winkler L, Chu Q, Loh KH, Corpuz EO, et al. A human microprotein that interacts with the mRNA decapping complex. Nat Chem Biol. 2017;13:174–80. 27918561 10.1038/nchembio.2249 PMC5247292

[134] Yang L, Tang Y, He Y, Wang Y, Lian Y, Xiong F, et al. High Expression of LINC01420 indicates an unfavorable prognosis and modulates cell migration and invasion in nasopharyngeal carcinoma. J Cancer. 2017;8:97–103. 28123602 10.7150/jca.16819 PMC5264044

[135] Godet Y, Moreau-Aubry A, Guilloux Y, Vignard V, Khammari A, Dreno B, et al. MELOE-1 is a new antigen overexpressed in melanomas and involved in adoptive T cell transfer efficiency. J Exp Med. 2008;205:2673–82. 18936238 10.1084/jem.20081356 PMC2571940

